# 3D Quantitative-Amplified Magnetic Resonance Imaging (3D q-aMRI)

**DOI:** 10.3390/bioengineering11080851

**Published:** 2024-08-20

**Authors:** Itamar Terem, Kyan Younes, Nan Wang, Paul Condron, Javid Abderezaei, Haribalan Kumar, Hillary Vossler, Eryn Kwon, Mehmet Kurt, Elizabeth Mormino, Samantha Holdsworth, Kawin Setsompop

**Affiliations:** 1 Department of Electrical Engineering, Stanford University, Stanford, CA 94305, USA; 2 Department of Neurology & Neurological Sciences, Stanford University, Stanford, CA 94305, USA; kyounes1@stanford.edu (K.Y.); hvossler@stanford.edu (H.V.); bmormino@stanford.edu (E.M.); 3 Department of Radiology, Stanford University, Stanford, CA 94305, USA; wangnx@stanford.edu; 4 Mātai Medical Research Institute, Tairāwhiti-Gisborne 4010, New Zealand; p.condron@matai.org.nz (P.C.); e.kwon@auckland.ac.nz (E.K.); s.holdsworth@matai.org.nz (S.H.); 5 Department of Mechanical Engineering, University of Washington, Seattle, WA 98195, USA; javidab@uw.edu (J.A.); mkurt@uw.edu (M.K.); 6 General Electric Healthcare, Tairāwhiti-Gisborne 4010, New Zealand; haribalan.kumar@ge.com; 7 Auckland Bioengineering Institute, University of Auckland, Auckland 1010, New Zealand; 8 Department of Anatomy and Medical Imaging—Faculty of Medical and Health Sciences & Centre for Brain Research, University of Auckland, Auckland 1010, New Zealand

**Keywords:** 3D amplified MRI, brain pulsatile motion, cerebrospinal fluid (CSF), cardiac gated cine MRI, Alzheimer’s disease (AD)

## Abstract

Amplified MRI (aMRI) is a promising new technique that can visualize pulsatile brain tissue motion by amplifying sub-voxel motion in cine MRI data, but it lacks the ability to quantify the sub-voxel motion field in physical units. Here, we introduce a novel post-processing algorithm called 3D quantitative amplified MRI (3D q-aMRI). This algorithm enables the visualization and quantification of pulsatile brain motion. 3D q-aMRI was validated and optimized on a 3D digital phantom and was applied *in vivo* on healthy volunteers for its ability to accurately measure brain parenchyma and CSF voxel displacement. Simulation results show that 3D q-aMRI can accurately quantify sub-voxel motions in the order of 0.01 of a voxel size. The algorithm hyperparameters were optimized and tested on *in vivo* data. The repeatability and reproducibility of 3D q-aMRI were shown on six healthy volunteers. The voxel displacement field extracted by 3D q-aMRI is highly correlated with the displacement measurements estimated by phase contrast (PC) MRI. In addition, the voxel displacement profile through the cerebral aqueduct resembled the CSF flow profile reported in previous literature. Differences in brain motion was observed in patients with dementia compared with age-matched healthy controls. In summary, 3D q-aMRI is a promising new technique that can both visualize and quantify pulsatile brain motion. Its ability to accurately quantify sub-voxel motion in physical units holds potential for the assessment of pulsatile brain motion as well as the indirect assessment of CSF homeostasis. While further research is warranted, 3D q-aMRI may provide important diagnostic information for neurological disorders such as Alzheimer’s disease.

## 1. Introduction

Changes in blood vessel pulsation and cerebrospinal fluid dynamics cause cyclic deformation of the brain [1,2,3,4,5,6]. Such brain motion can be altered by neurological pathologies such as hydrocephalus [7,8,9], Chiari I malformation [10,11,12], idiopathic intracranial hypertension [13,14], and age-related diseases in small cerebral vessels [15,16,17]. Furthermore, the glymphatic system [18], responsible for the macroscopic waste clearance of the central nervous system, is thought to depend on CSF pulsation in the perivascular pathways to remove metabolic waste from the brain [19,20]. Quantifying the cardiac- and CSF-induced (pulsatile) brain motion *in vivo* may therefore provide valuable insights into neurological pathologies and their effects on the brain’s biomechanical response, making it a useful tool for research, and it has the potential to assist in the diagnosis and management of such diseases.

Various MRI techniques are available that enable the visualization and quantification of pulsatile brain motion. Phase contrast MRI (PC-MRI) [3,21,22,23] uses bipolar gradients to measure tissue velocity over the cardiac cycle in different directions, which can lead to longer scanning times. To estimate the displacement, the measured velocities need to be integrated over time, which can introduce additional errors. Complementary Spatial Modulation of Magnetization (CSPAMM) [3,24,25] measures tissue displacement by tagging or nulling the signal in the region of interest. This technique has demonstrated an ability to measure brain motion in the cranial–caudal direction [25]. However, due to this technique’s low spatial resolution, it may pose difficulty in detecting small brain motion in 3D within a clinically feasible scan time. Displacement encoding with stimulated echoes (DENSE) MRI [5,6,26,27,28] is a technique that can measure tissue displacement with high spatial and temporal resolution and is sensitive to small displacements (0.01 mm). However, it requires encoding in different directions and suffers from a low SNR, both of which translate to long scan times.

Amplified MRI (aMRI) [29,30] is a relatively new method that can visualize pulsatile brain tissue motion over the cardiac cycle by amplifying subtle temporal intensity changes in cardiac gated ‘cine’ MRI data. aMRI offers several advantages over other techniques, including pronounced tissue contrast (when coupled with the balanced steady-state free precession (bSSFP) MRI sequence), a short scan time (it does not require displacement encoding in multiple directions), and a higher spatial and temporal resolution compared to DENSE-MRI.

Additionally, the ability of aMRI to reveal mesoscopic brain deformations makes it a promising tool for assessing various neurological disorders, including Chiari Malformation [30,31], aneurysms (aFlow [32,33]), concussion [34], and acute hemorrhagic stroke [35]. Recently, aMRI was extended to three dimensions, enabling the visualization of brain motion in all three planes (3D aMRI [36,37]). Compared to 2D acquisition, 3D aMRI enhances image quality and improves the visualization of pulsatile brain motion by capturing out-of-plane motion (third direction). The high spatial and temporal resolution of the underlying cine acquisition, along with the conspicuous brain tissue and CSF contrast enabled by the underlying bSSFP sequence, allows for the observation of the brain’s biomechanical response in exquisite detail.

However, 3D aMRI still lacks the ability to quantify the sub-voxel motion field in physical units. Our study introduces a novel 3D quantitative aMRI (3D q-aMRI) post-processing algorithm that enables the visualization and quantification of pulsatile sub-voxel brain motion by applying it to 3D bSSFP cine data. The new 3D q-aMRI is an extension of the original 3D aMRI algorithm and is based on solving the optical flow equation over the coefficients of the 3D complex linear steerable pyramid decomposition [38,39,40].

To validate the developed algorithm, we tested 3D q-aMRI on a 3D digital phantom to assess its behavior and limitations. *in vivo* validation was performed to fine-tune the algorithm hyperparameters and to test the ability of the algorithm to quantify sub-voxel displacement motion observed in 3D aMRI. The results from 3D q-aMRI were qualitatively compared to phase-contrast MRI (PC-MRI), the conventional technique for capturing brain motion. The repeatability and reproducibility of 3D q-aMRI were evaluated in six healthy volunteers using a scan–rescan approach. Finally, preliminary 3D q-aMRI data were acquired on nine participants: four healthy controls and five with confirmed neurodegenerative disorders. Our aim was to test the potential diagnostic value of 3D q-aMRI in identifying disease-induced biomechanical differences.

## 2. Materials and Methods

### 2.1. Human Subjects

Experiments were conducted under ethical approval from The New Zealand Health and Disability Ethics Committee and Stanford University. Thirteen healthy adult volunteers were scanned (eight males and five females between 21 and 37 years old), with eight repeated scans acquired on six of these volunteers within the same session. Data were also acquired from nine subjects: four healthy controls (aged 55–75 years; one male and three females) and five individuals with confirmed neurodegenerative disorders. These included two patients with mild cognitive impairment (MCI) due to dementia (a 70-year-old female and a 74-year-old male), two patients with dementia due to Alzheimer’s disease (AD) (aged 62 years and 85 years), and one 76-year-old male with Lewy body dementia (LBD).

### 2.2. MRI Acquisition

A 3T MRI scanner (SIGNA Premier; GE Healthcare, Milwaukee, WI, USA) was used with an AIR™ 48-channel head coil. Three-dimensional volumetric cardiac-gated cine (bSSFP/FIESTA) MRI datasets were acquired as follows: Sagittal plane, FOV = 24 × 24 cm^2^, matrix size = 200 × 200, TR/TE/flip-angle = 2.8 ms/1.4 ms/25°, acceleration factor = 8, resolution = 1.2 mm isotropic, peripheral pulse gating with retrospective binning to 20 cardiac phases, 120 slices for whole brain coverage, and a scan time of 2:30 min.

For segmentation purposes, we also collected T1 MPRAGE volumetric data from the six volunteers who were scanned for repeatability and reproducibility analyses. The following parameters were used: Sagittal plane, FOV = 24 × 24 cm^2^, matrix size = 200 × 200, TR/TE/flip-angle = 2500 ms/2.4 ms/8°, resolution = 1.2 mm isotropic.

For comparison to past studies using the common phase contrast MRI (PC-MRI) approach, we also collected a cine PC-MRI dataset on two volunteers, with the following parameters: FOV = 24 × 24 cm^2^, velocity encoding = 0.5 cm/s, matrix size = 128 × 128 (zero-filling to 200 × 200), slice thickness = 4 mm, and TR/TE/flip-angle = 27 ms/14 ms/10°. Velocity encoding was performed in the right–left direction in the axial plane, and in the superior–inferior direction for the sagittal and coronal planes.

PET/MR scans were performed using a simultaneous time-of-flight (TOF)-enabled PET/MR scanner (3T SIGNA Premier; GE Healthcare, Milwaukee, WI), with high sensitivity (23.3 cps/kBq). MRI data were collected simultaneously with the PET data from both healthy controls and participants with dementia using the same aMRI parameters as previously mentioned.

### 2.3. Motion Estimation

**Figure 1:**
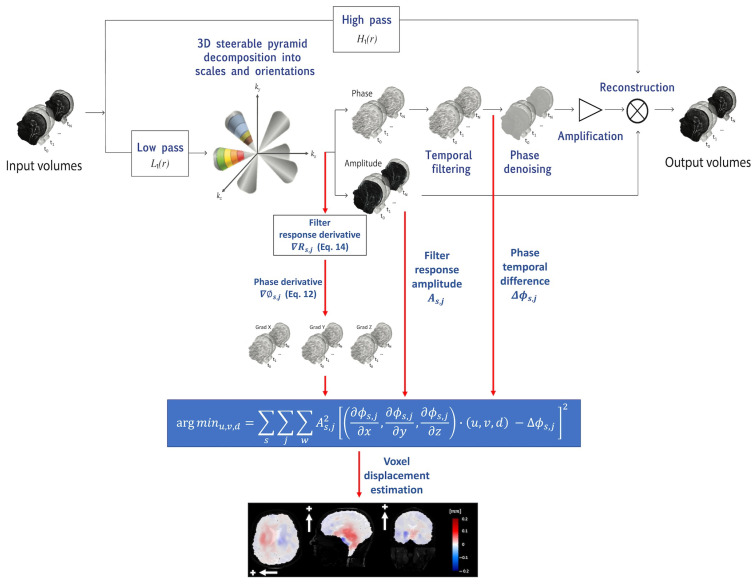
The 3D q-aMRI algorithm pipeline begins with the decomposition of volumetric cine MRI using the 3D complex steerable pyramid. This process separates the images into various scales and orientations, isolating different spatial frequency components. The decomposed images are then split into amplitude and phase components, with the phases encoding information about sub-voxel motion. Next, the phase components are temporally filtered at each spatial location, orientation, and scale to enhance significant temporal changes. These filtered phases are split and proceed along two paths: the original amplification path for visualization and the quantification path for generating voxel displacement maps. For quantitative estimation, the data undergo the estimation of the spatial phase derivative. This involves estimating the spatial phase derivative from the decomposed image. The voxel displacement field is then calculated by solving a least squares optimization objective. This formula calculates the best-fit voxel displacement field that aligns the phase derivatives with the phase temporal changes. The color-coded images display the estimated voxel displacements in the axial (L/R direction, white arrow), sagittal (S/I direction, white arrow), and coronal (S/I direction, white arrow) planes. The plus sign indicates the positive direction of motion.

3D q-aMRI is a natural extension of the original 3D aMRI algorithm (Figure 1). As such, the algorithm starts by decomposing the brain volumetric ‘cine’ MRI data using the 3D steerable pyramid decomposition [36,37]. The 3D steerable pyramid is a complex linear multiscale and multi-orientation decomposition in which steerable filters are used in a recursive scheme (low-pass filtering and downsampling) [41,42]. The scales basis functions are bandpass filters in the frequency domain. They are calculated in polar coordinates as a multiplication of low- and high-pass filters. The low-pass and high-pass filters for each scale are given by the following equations:

(1) $$H_{s}\left(r\right)=\left\{\begin{array}{cc} 1, & \frac{r}{s}\geq 1\\ \left|cos\left(\frac{\pi }{2}log_{2}\left(\frac{r}{s}\right)\right)\right|, & 0.5<\frac{r}{s}<1\\ 0, & 0<\frac{r}{s}\leq 0.5 \end{array}\right.$$

(2) $$L_{s}\left(r\right)=\sqrt{1-H_{s}^{2}\left(r\right)}=\left\{\begin{array}{cc} 0, & \frac{r}{s}\geq 1\\ \left|sin\left(\frac{\pi }{2}log_{2}\left(\frac{r}{s}\right)\right)\right|, & 0.5<\frac{r}{s}<1\\ 1, & 0<\frac{r}{s}\leq 0.5 \end{array}\right.$$

where $r=\sqrt{k_{x}^{2}+k_{y}^{2}+k_{z}^{2}}$ and *s* represents the level scale. The bandpass filter of each level is given by the following:

(3) $$B_{s}\left(r\right)=H_{s}\left(r\right)\times L_{s-1}\left(r\right)$$

The angular (orientation) filters are 3D cones oriented along the six vertices of cuboctahedron (Table 1) and satisfy the following equation in the frequency domain:

(4) $$B_{j}\left(k_{x},k_{y},k_{z}\right)=\frac{{\left(\alpha_{j}k_{x}+\beta_{j}k_{y}+\gamma_{j}k_{z}\right)}^{2}}{k_{x}^{2}+k_{y}^{2}+k_{z}^{2}},\;j=1,2,\ldots ,6$$

where $\alpha_{j},\beta_{j},\gamma_{j}$ are the directions of the axes of symmetry of the six basis filters $B_{j}$. The 3D steerable filters are constructed as follows:

(5) $$f_{s,j}\left(k_{x},k_{y},k_{z}\right)=B_{s}\left(\sqrt{k_{x}^{2}+k_{y}^{2}+k_{z}^{2}}\right)\times B_{j}\left(k_{x},k_{y},k_{z}\right)$$

and the final image response is calculated as follows:

(6) $$R_{s,j}\left(x,y,z\right)=F^{-1}\left\{F\left\{I(x,y,z)\right\}\times f_{s,j}\right\}=A_{s,j}\cdot e^{i\phi_{s,j}}$$

where *s* and *j* are the scaling factor and orientation direction respectively; $F\left\{I(x,y,z)\right\}$ is the Fourier transform of the image; and $F^{-1}$ is the inverse Fourier transform. The decomposition outputs a complex number with amplitude ($A_{s,j}$) and phase ($\phi_{s,j}$) at each scale and orientation.

The phases of the decomposition encode information about the sub-voxel motion, similar to the Fourier shift theorem. This information is exploited to achieve both magnification (visualization) and quantification of the voxel displacement field. The phases are separated from the amplitude component and temporarily bandpass filtered to isolate the desired frequency range, and to remove any DC component.

The bandpass phases proceed through two distinct processing pathways: (a) the original 3D aMRI algorithm [36], which includes phase denoising using an amplitude-weighted Gaussian smoothing filter, multiplication by the amplification factor α, and reconstruction that results in motion magnification, and (b) the new voxel displacement quantification algorithm. The quantification algorithm is based on solving the optical flow equation, which assumes the brightness constancy constraint (that the brightness of a moving point in an image remains constant over time). However, there is a subtle distinction from the standard optical flow approach that we use here. The equation is instead solved over the filtered phases of the coefficients of the 3D complex-valued steerable pyramid decomposition. Here, we are guided by the work from Fleet and Jepson [38], who showed the general case whereby the temporal evolution of (spatial) contours with constant phase in the image subbands (such as used in a complex wavelet decomposition) correspond to the motion field. Mathematically, this can be written as follows:

(7) $$\begin{array}{cc} \phi_{s,j}\left(x,y,z,0\right) & =\phi_{s,j}\left(x-u\left(x,y,z,t\right),y-v\left(x,y,z,t\right),\right.\\ \; & \;\left.z-d\left(x,y,z,t\right),t\right) \end{array}$$

Here, $\phi_{s,j}$ are the phases of the 3D complex steerable pyramid, and (u,v,d) are the voxel displacement fields we would like to estimate. We can expand the right-hand side using the Taylor series around $\left(x,y,z\right)$ to obtain the following:

(8) $${\Delta \phi }_{s,j}=\left(\frac{\partial \phi_{s,j}}{\partial x},\frac{\partial \phi_{s,j}}{\partial y},\frac{\partial \phi_{s,j}}{\partial z}\right)\cdot \left(u,v,d\right)+O\left(u^{2},v^{2},d^{2}\right)$$

where ${\Delta \phi }_{s,j}\left(x,y,z,t\right)=\phi_{s,j}\left(x,y,z,t\right)-\phi_{s,j}\left(x,y,z,0\right)$ and $O\left(u^{2},v^{2},d^{2}\right)$ are higher-order terms in the Taylor expansion. The main assumption here is that the motion is at the sub-voxel level, and as a result, the higher-order terms in the Taylor expansion are negligible. Therefore, the solution can be approximated using only the linear term:

(9) $${\Delta \phi }_{s,j}=\left(\frac{\partial \phi_{s,j}}{\partial x},\frac{\partial \phi_{s,j}}{\partial y},\frac{\partial \phi_{s,j}}{\partial z}\right)\cdot \left(u,v,d\right)$$

We solve this linear equation using a weighted least squares objective function over the various orientations and scales of the 3D steerable pyramid decomposition [39] as follows:

(10) $$\begin{array}{cc} \underset{u,v,d}{\arg\;\min}\sum_{s}\sum_{j}\sum_{W} & A_{s,j}^{2}\left[\left(\frac{\partial \phi_{s,j}}{\partial x},\frac{\partial \phi_{s,j}}{\partial y},\frac{\partial \phi_{s,j}}{\partial z}\right)\cdot \left(u,v,d\right)\right.\\ \; & \;\;\;\;-{\left.\Delta \phi_{s,j}\right]}^{2} \end{array}$$

where *W* is a Gaussian window (which assumes that the motion field is smooth around a given voxel); $\phi_{s,j}\;$ and $A_{s,j}$ are the phase and amplitude of the output of the filters in the 3D steerable pyramid decomposition; and ${\Delta \phi }_{s,j}$ is the phase difference between the frames $t_{n}$ and $t_{0}$. Note that the extent of the Gaussian window is calculated according to the standard deviation (σ) value using the following equation:

(11) $$WindowSize=2*\left(2*\sigma \right)+1$$

and that throughout this work, we use a Gaussian window with σ=5, which equates to a support window of 21×21×21 pixels. In addition, we notice that phases with orientations corresponding to edges—which are more reliable for motion estimation—will have a larger weighting term $A_{s,j}^{2}$.

Furthermore, conventional discrete derivative operators (e.g., the intermediate or the central kernels) are sensitive to noise when estimating derivatives in high spatial frequency images, such as in the filter responses of the higher levels of the 3D steerable pyramid. Therefore, to increase the algorithm’s robustness to noise and improve the estimated motion field accuracy, the phases’ spatial derivatives $\left(\frac{{\partial \phi }_{s,j}}{\partial x},\frac{{\partial \phi }_{s,j}}{\partial y},\frac{{\partial \phi }_{s,j}}{\partial z}\right)$ were estimated using the following approach [38]:

(12) $$\nabla \phi_{s,j}\left(\vec{x},t\right)=\;\frac{Im\;\left[R_{s,j}^{*}\;\left(\vec{x},t\right)\;\cdot \;\nabla R_{s,j}\left(\vec{x},t\right)\right]}{A_{s,j}^{2}\left(\vec{x},t\right)}$$

where $R_{s,j}\left(\vec{x},t\right)$ is the image response for the s,jth filter (complex number) and Im is the imaginary operator. $\nabla R_{s,j}\left(\vec{x},t\right)$ still contains components with high spatial frequencies, but it can be demodulated as follows, with the image response expressed as:

(13) $$R_{s,j}\left(\vec{x},t\right)=M\left(\vec{x},t\right)\cdot e^{i\vec{x}k_{0}}$$

where $k_{0}$ is the peak tuning frequency of the corresponding subband’s filter. Taking the derivative of $R_{s,j}\left(\vec{x},t\right)$ results in the following:

(14) $$\nabla R_{s,j}\left(\vec{x},t\right)=\nabla M\left(\vec{x},t\right)\cdot e^{i\vec{x}k_{0}}+ik_{0}\cdot R_{r,\theta }\left(\vec{x},t\right)$$

where $M\left(\vec{x},t\right)$ is the demodulated filter response, which contains only low spatial frequencies. Finally, we notice that traditional derivative filters are often inappropriate for multi-dimensional problems [43]; therefore, $\nabla M\left(\vec{x},t\right)$ was approximated using the following derivative filters:

$$Derivative=\left[0.109604,0.276691,0,-0.276691,-0.109604\right]$$

$$PreFilter=\left[0.037659,0.249153,0.426375,0.249153,0.037659\right]$$

where the *Derivative* filter is applied in the desired direction, and the *PreFilter* is applied in the two remaining directions. In comparison to the traditional intensity-based optical flow algorithms, which estimate motion larger than one voxel, this approach of using image local phase information can estimate sub-voxel motion on the order of 0.01 pixel size [39,44].

### 2.4. Digital Phantom Simulation

**Figure 2:**
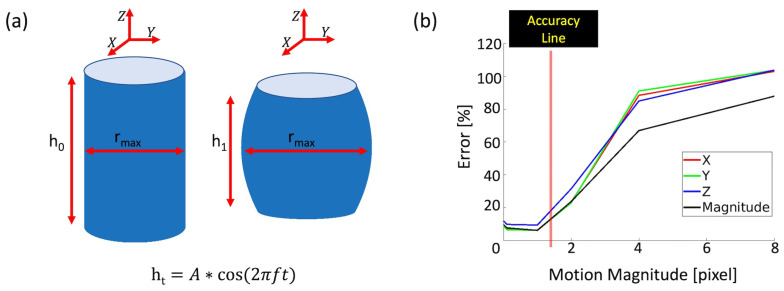
Validation of 3D q-aMRI on a 3D cylinder phantom (initial height $h_{0}$ and $r_{0}$ radius) that undergoes cyclic tension and compression. (**a**) The phantom at reference time $t_{0}$ and deformation time $t_{i}$. (**b**) Error as a function of displacement in the absence of noise.

To evaluate the performance of 3D q-aMRI, a 3D digital phantom was used to mimic the subtle deformation, intensity, and contrast of the lateral ventricles observed in 3D aMRI (Supplemental Video S1). The phantom consists of a 3D cylinder with an initial height $h_{0}$ and an initial radius $r_{0}$ and was simulated using MATLAB (MathWorks, Natick, MA, version 2022b). The two ends of the 3D cylinder were deformed according to the equation $h_{t}=A*cos\left(2\pi ft\right)$, resulting in cyclic compression and tension along its axis (Figure 2a). We made the following assumptions:

1. The phantom volume remains constant over time, resulting in expansion and compression along the cylinder’s radius.
2. The radius follows a parabolic curve, $r\left(z\right)=\;a*z^{2}+\;r_{max}$, where $r_{max}$ is the maximum radius and *z* is the position along the axis of the cylinder of height $h_{t}$. These assumptions result in the following equation for *a* and $r_{max}$:

(15) $$\frac{h_{t}^{5}}{5}a^{2}\;+\frac{2*h_{t}^{3}*r_{max}}{3}a+\;h_{0}*r_{0}^{2}+\;h_{t}*r_{max}^{2}\;\;=\;0$$

Here $r_{max}=\frac{r_{0}}{2}\left(\sqrt{25-30*\left(1-\frac{h_{t}}{h_{0}}\right)}-5\right)$, which was selected as in our previous 3D aMRI work [37]. This choice of $r_{max}$ ensures that the phantom undergoes sub-voxel motions.

The estimated motion field was evaluated against the true motion field using the following metrics:

(1) Pearson’s Linear Correlation
(2) $Error\left[\%\right]=Mean\left[abs\;\left[\frac{x_{estimated}\;-\;\;x_{true}\;}{x_{true}}\right]*\;100\right]\left[\%\right]$
(3) Ninety-ninth percentile of the error distribution

The following were evaluated: (1) the algorithm’s accuracy as a function of the magnitude of the true motion (Figure 2b); (2) the algorithm’s robustness to various Gaussian noise levels (Table 2); and (3) the effects of the algorithm’s hyperparameters (number of pyramid levels in the least squares, temporal filtering, and Gaussian window σ parameter) and various temporal frequency motions on the estimated motion field (Supplemental Figures S1–S4).

### 2.5. In Vivo Validation

First, it is important to note that we undertook the following standardization procedures:

(1) Since the native image size of the acquisitions determines the extent and resolution of the 3D steerable pyramid filters, we ensured that all *in vivo* datasets were zero-padded to a uniform dimension of 256 × 256 × 256.
(2) Accordingly, for the analysis of different image resolutions, we used the corresponding σ values to match the physical extent of the Gaussian smoothing filter. For example, to compare a 2.4 mm isotropic resolution (128 × 128 × 128) versus an isotropic 1.2 mm isotropic resolution (256 × 256 × 256) dataset (the latter with σ=5), we first zero-padded the 2.4 mm dataset and used σ=2.5 to ensure the filter has the same extent as the 1.2 mm dataset in physical units.
(3) To generate a sharp frequency response of the temporal filter that will pass the desired frequency band, the number of cardiac phases was extended to 5 periods—which is a valid procedure since the motion of the cine data is periodic. This was performed since the native number of cardiac phases of 20 may generate side lobes in the filter frequency spectrum—which in turn may propagate errors in the voxel displacement estimation due to noise.

**Figure 3:**
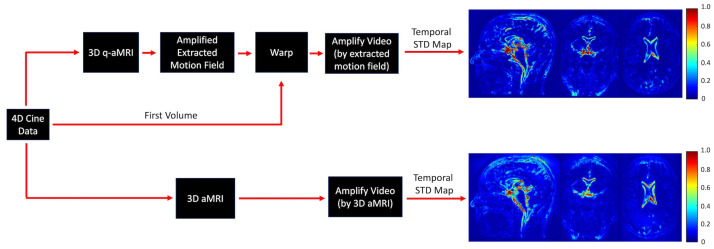
*In vivo* validation of the 3D q-aMRI against the observed signal in 3D aMRI. The 4D cine data are amplified by 3D aMRI. In addition, the first volume in the cine data is warped by an amplified version of the estimated motion field, and normalized temporal variance maps are calculated for both amplified movies. The maps suggest that 3D q-aMRI quantification output matches the motion observed qualitatively in 3D aMRI.

To validate the ability of 3D q-aMRI to accurately quantify the observed motion in 3D aMRI, we followed three steps. First, 3D aMRI was used to amplify the signal in the 4D *in vivo* data. Then, the voxel displacement field was calculated using 3D q-aMRI, and a second amplified video was created by warping the first volume in the data according to an amplified version of the estimated motion field. Next, normalized temporal standard deviation maps were calculated for both amplified videos (Figure 3), and these were compared qualitatively.

Lastly, we also tested and optimized the algorithm’s hyperparameters (number of pyramid levels, temporal filtering, and Gaussian window σ parameter) for the *in vivo* data. Lastly, the effect of varying image resolution on the estimated voxel displacement field was tested.

### 2.6. In Vivo Analysis

An *in vivo* analysis was conducted to provide insights into the performance and potential applications of 3D q-aMRI in biomedical research and clinical practice. First, we qualitatively compared 3D q-aMRI against ground truth phase contrast (PC)-MRI data. The velocities were integrated over time to extract the displacement field. The motion profile was also evaluated in the through-plane direction through the cerebral aqueduct. Second, the repeatability and reproducibility of 3D q-aMRI were assessed using 4 scans and 4 rescans (for a total of 8 scans) in 6 healthy volunteers. For each volunteer, the following analyses were conducted: initially, the MPRAGE data were registered to the first ‘cine’ bSSFP scan using rigid body registration in MATLAB (MathWorks, Natick, MA, USA, 2022). Second, the registered MPRAGE data were used to segment [45] the following brain regions: lateral ventricles, the 3rd ventricle, the 4th ventricle, the brainstem, and the cerebellum. Next, the voxel displacement field was extracted for each scan using 3D q-aMRI. Each scan (of the eight) was registered to the first scan using rigid body registration, and the extracted transformation was applied to each of the voxel displacement fields. Lastly, the average motion profile in the superior and inferior directions throughout the cardiac cycle was extracted for each of the segmented brain regions. In addition, statistical analysis was conducted as follows: For each brain region and subject, the distance between the average signal (computed over the eight scans) and each individual measurement was computed using dynamic time warping. Dynamic Time Warping (DTW) is a technique used to measure the similarity between two time series by warping their time axes to achieve the best match between corresponding elements. This alignment minimizes the distance between the sequences. The calculated distances were used to calculate the Intraclass Correlation Coefficient (ICC). Finally, 3D q-aMRI data were acquired on patients with dementia and a healthy age-matched control to assess the potential diagnostic value in identifying disease-induced biomechanical differences. Note that this is an early preliminary test, suggesting that, regardless of the results of these scans, further research will be necessary to determine the technique’s potential as a diagnostic tool.

## 3. Results

### 3.1. Phantom Simulations

Figure 2b illustrates the error of the estimated motion field as a function of the motion magnitude in the X, Y, and Z directions, in the absence of noise. The algorithm is accurate for sub-voxel motion as small as 0.0001 of a pixel in size but breaks down for large motions > 1.5 pixels. Table 2 shows that with increasing Gaussian noise, 3D q-aMRI can accurately quantify motion > 0.005 pixels while remaining robust to noise. voxel displacement can be accurately extracted for an SNR as low as 25, maintaining high correlation (0.95), low error (13%), and relatively small error distribution tail (99% of the error is below 30%). In addition, higher noise levels require a larger spatial Gaussian window σ parameter for accurate voxel displacement estimation at the cost of spatial smoothing. Notice that these results represent an ideal limit and that, in practice, there are other sources of ‘noise’ that were not simulated in the digital phantom.

Supplemental Figures S1–S4 demonstrate the effects of the algorithm’s hyperparameters on its performance. Supplemental Figure S1 shows the estimated field error as a function of the number of pyramid levels used to solve the weighted least squares equation. The results depend on the type of motion, whether it be local deformation or global translation. For global translation, using all pyramid levels results in better accuracy, whereas for local deformation, only the two highest levels of the pyramid are required. We know that the human brain exhibits localized deformation; as such, as will be seen in the *in vivo* section, only the first two levels of the 3D steerable pyramid are required for the accurate estimation of the voxel displacement field.

Supplemental Figure S2 displays the effect of temporal filtering on the estimated motion field for an SNR of 25. As shown, prior knowledge of the expected motion (temporal frequencies present in the data) has a significant impact on the accuracy of the estimated voxel displacement field. We utilize the fact that our data are cardiac-gated and filter out temporal frequencies outside the one to four heart rate harmonics when estimating the voxel displacement field. We will provide further justification for this choice in the *in vivo* analysis section.

Supplemental Figure S3 shows the effect of varying the Gaussian standard deviation on the estimated motion field for an SNR of 25. As observed, increasing the standard deviation leads to a better estimation field up to a certain threshold, after which the estimated field remains almost unchanged. We used this observation to determine the optimized standard deviation for the *in vivo* data, as will be seen in the *in vivo* validation section.

Finally, Supplemental Figure S4 demonstrates that the algorithm can adequately extract the sub-voxel displacement field for motion with a combination of different temporal frequencies. This capability will be further discussed in the section comparing 3D q-aMRI and PC-MRI.

### 3.2. In Vivo Validation

**Figure 4:**
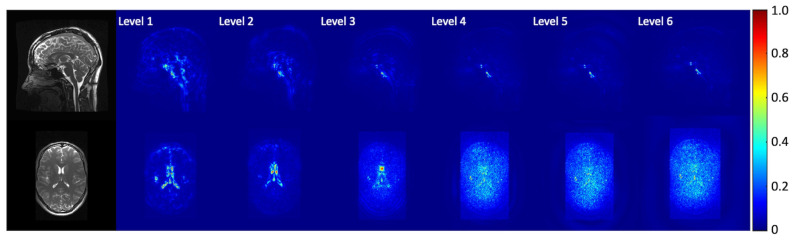
Normalized temporal standard deviation maps of the amplified videos for different pyramid levels. The data were amplified with an amplification parameter of 30 with a Gaussian window with σ=5. Coherent motion exists mainly in the first two levels of the steerable pyramid.

**Figure 5:**
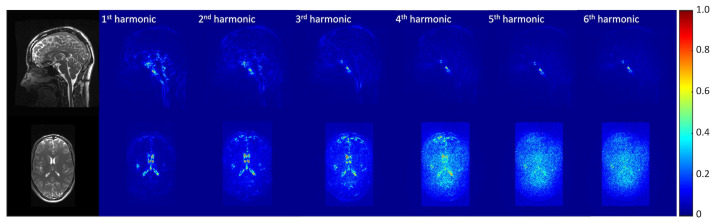
Normalized temporal standard deviation maps of the amplified videos for different temporal frequency bands. The data were amplified with an amplification parameter of 30 with a Gaussian window with σ=5. Motion was extracted using the first two levels of the steerable pyramid. Coherent motion exists mainly in the one to four heart rate harmonics band.

**Figure 6:**
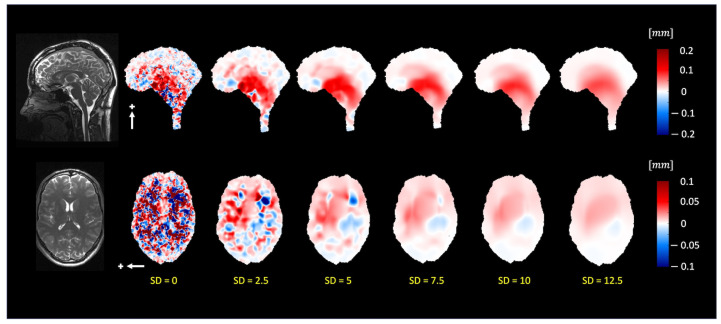
The pulsatile brain motion in the sagittal (S/I direction, indicated by a white arrow) and axial (L/R direction, indicated by a white arrow) for different standard deviation sizes of the Gaussian window. The Gaussian smoothing reduces the noise level in the estimated motion field. For standard deviations larger than σ=5, the estimated motion field is smooth and generally remains constant.

Figure 3 demonstrates that 3D q-aMRI successfully quantified the sub-voxel motion observed in the 3D aMRI (Supplemental Video S2) using the optimized algorithm’s hyperparameters. These parameters include bandpass-filtering (one to four heart rate harmonics) the phases and solving the weighted least squares using only the first two highest levels of the 3D steerable pyramid with a Gaussian window of σ=5 and a support window of 21×21×21 pixels. The justification for these choices is provided in Figure 4, Figure 5 and Figure 6 and Supplemental Videos S3–S5.

Figure 4 depicts the normalized temporal standard deviation maps of the amplified (3D aMRI) videos (Supplemental Video S3) for different pyramid levels (levels 1–6). Most of the motion information is observed within the first two levels of the 3D steerable pyramid.

Figure 5 shows the normalized temporal standard deviation maps of the amplified (3D aMRI) videos (Supplemental Video S4) for different temporal frequency bands, demonstrating that motion information exists within the one to four heart rate harmonics band.

Figure 6 depicts the extracted motion field by 3D q-aMRI (Supplemental Video S5) for varying Gaussian windows (σ ranging from 0 to 12.5). The estimated field profile is almost unchanged for σ>5. For these reasons, the algorithm’s hyperparameters were chosen as mentioned above.

**Figure 7:**
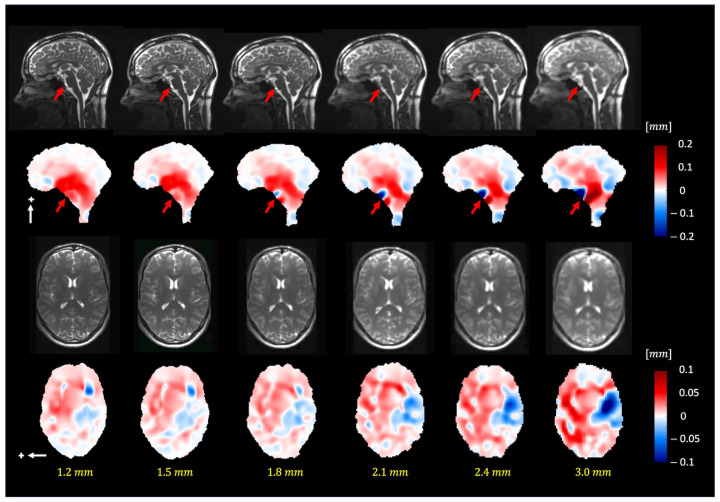
The pulsatile brain motion in the sagittal (S/I direction, indicated by a white arrow) and axial (L/R direction, indicated by a white arrow) directions for different isotropic spatial resolutions. Plus sign represent the positive direction of motion. As can be seen, the algorithm can robustly estimate the motion field for different image resolutions (up to 1.8 mm isotropic voxel size). Note that the dark blue/red regions (red arrows) in the sagittal plane point to the basilar artery, which exhibits apparent motion (larger than 1.5 pixels).

Figure 7 depicts the extracted motion field by 3D q-aMRI (Supplemental Video S6) for different isotropic voxel sizes (1.2–3.0 mm^3^). The extracted motion field remains consistent up to an isotropic voxel size of 1.8 mm. The red arrows in the sagittal plane point to the basal artery (Supplemental Video S7), which exhibits apparent motion (larger than 1.5 pixels) and, based on the phantom simulations, will result in an error in the estimated motion field.

### 3.3. In Vivo Analysis and Repeatability Study

**Figure 8:**
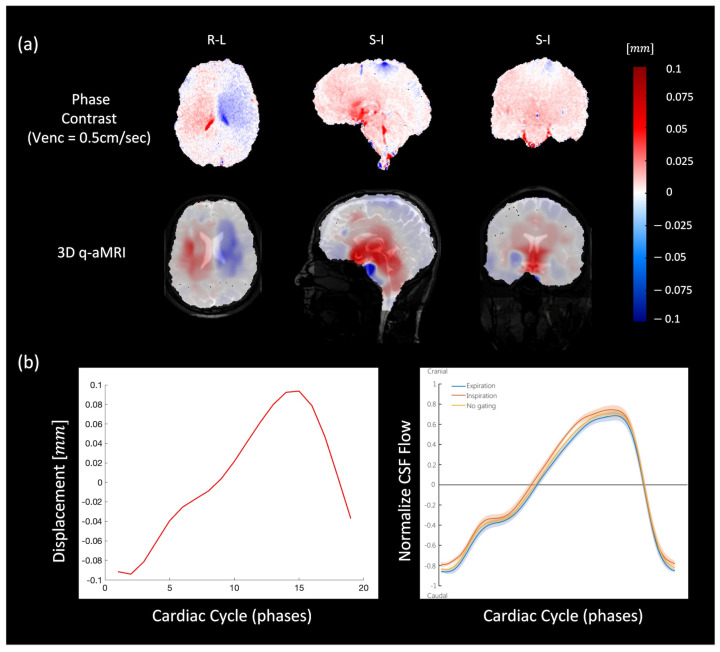
(**a**) Comparison between PC-MRI (top) and 3D q-aMRI (bottom) for sagittal (S/I direction), coronal (S/I direction), and axial (L/R direction) planes. The estimated field captures the relative brain tissue deformation over time and the physical change in shape of the ventricles by the relative movement of the surrounding tissues. (**b**) The extracted flow/motion profile through the cerebral aqueduct as extracted by 3D q-aMRI (left), which is comparable to that reported by [46] as shown in the inset (right). Note that [46] the graph seen here is normalized, but the actual CSF flow values reported were an order of magnitude higher than the 3D q-aMRI flow profile.

In both 3D q-aMRI and cine PC-MRI, the general characteristics of brain motion were found to be similar and on the same scale (Figure 8a and Supplemental Video S8). In both sequences, the predominant tissue displacement was in the cranial–caudal direction in the sagittal and coronal planes, and expanding/contracting motion in the axial plane, with the largest brain tissue displacement occurring around the midbrain, cerebellar tonsils, brainstem, and hypothalamus. Minimal displacement occurred in the frontal lobe, parietal lobe, occipital lobe, temporal lobe, and posterior cerebellum. Figure 8b depicts the voxel displacement profile (throughout the cardiac cycle) in the superior–inferior direction through the cerebral aqueduct as captured by 3D q-aMRI. This motion/flow profile is consistent with other literature [46] and was achieved only with the optimized algorithm’s hyperparameters (Supplemental Figure S5).

**Figure 9:**
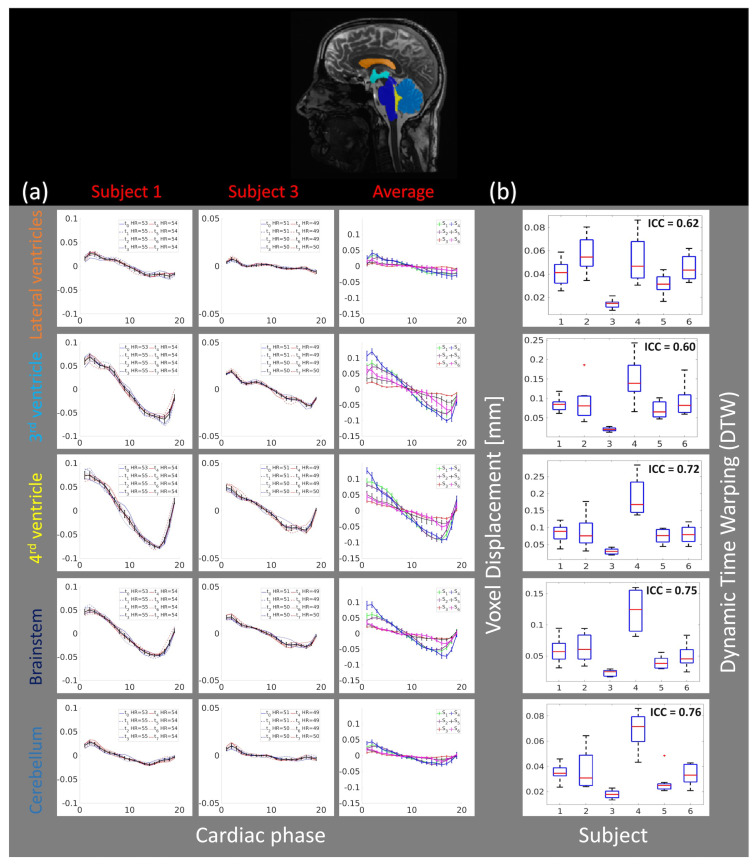
(**a**) The average (over different brain regions) voxel displacement profile for two subjects ($S_{1}$ and $S_{3}$) in the S/I direction for eight scans ($t_{0}$ to $t_{7}$). Top—the brain regions (lateral ventricles, 3rd ventricle, 4th ventricle, brainstem, and cerebellum) where the average voxel displacement was estimated. Bottom—the first two columns depict the voxel displacement profile for all scans, for each of the two subjects. The black line represents the average motion over all scans, together with an error bar (95% confidence interval). The last column depicts the average motion for all six subjects, along with error bars representing the 95% confidence interval. The results indicate high repeatability across the time points within each subject, with similar motion patterns but different magnitudes across all subjects. (**b**) The boxplots for each brain region and the Intraclass Correlation Coefficient (ICC) of the dynamic time warping (DTW) distance. The plus sign denotes an outlier.

Figure 9a depicts the repeatability and reproducibility of the voxel displacement brain motion throughout the cardiac cycle in five different brain regions (lateral ventricles, the 3rd ventricle, the 4th ventricle, the brainstem, and the cerebellum). The first two columns depict the brain motion in the superior inferior direction in the different brain regions throughout the cardiac cycle for two selected subjects ($S_{1}$ and $S_{3}$). The last column depicts the average motion (over all eight scans) and the error bar (95% confidence interval) for each brain region for all six subjects. Supplemental Figure S6 depicts the same results for all six subjects together with their Pearson correlation matrix. It is worth mentioning that in some of the repeated scans global flickering was present in raw cine data (Supplemental Video S9), which resulted in larger variability within the measurements ($S_{4}$ and $S_{5}$ in Supplemental Figure S6). Nevertheless, the results (Figure 9b) suggest that intra-subject variability is small compared to inter-subject variability, potentially indicating that 3D q-aMRI is sensitive to individual differences.

**Figure 10:**
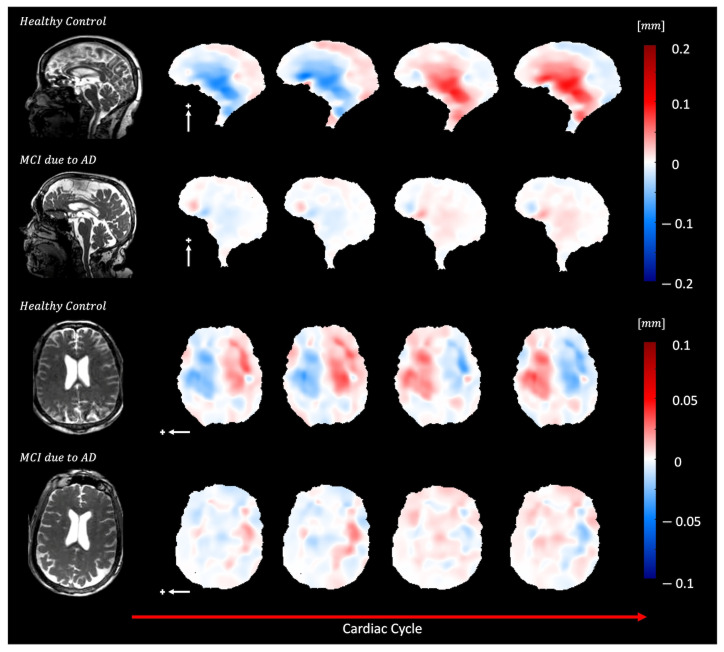
Depicts diffuse reduction in brain bulk displacement on both the sagittal (S/I direction, white arrow) and axial (L/R direction, white arrow) planes for elderly adults with MCI due to dementia (70-year-old female) compared to an elderly control (74-year-old female) plus sign represent the positive direction of motion. In addition, loss of symmetry and irregular lateral motion of the lateral ventricles are seen in the displacement maps.

Figure 10 and Supplemental Video S10 show brain motion in a healthy control and a subject with mild cognitive impairment (MCI) due to Alzheimer’s disease (AD). In the AD participant, a diffuse reduction in brain bulk displacement is observed in both the sagittal and axial planes. Additionally, the displacement maps show a loss of symmetry and irregular motion of the lateral ventricles.

Supplemental Video S11 illustrates brain motion in a healthy control and a subject with dementia due to AD. Compared to the healthy control, the motion around the lateral ventricles in the sagittal plane is abnormal in the AD participant. Additionally, in the axial view, the motion is irregular and asymmetric.

Supplemental Video S12 presents brain motion in a healthy control and a participant with dementia due to AD. Compared to the healthy control, the motion in the sagittal plane around the lateral ventricles is reduced and irregular in the participant with dementia.

Supplemental Video S13 displays brain motion in a healthy control and a participant with MCI due to AD. Compared to the healthy control, the motion around the lateral ventricles in the sagittal plane is abnormal in the MCI participant. In the axial plane, the motion is asymmetric and wobbly.

Supplemental Video S14 shows brain motion in a healthy control and a subject with Lewy body dementia (LBD). Compared to the healthy control, the motion in the sagittal plane around the aqueduct and fourth ventricle is abnormal in LBD cases, with motion occurring in the anterior-posterior (AP) direction instead of the superior-inferior (SI) direction. The displacement maps also reveal asymmetrical motion of the lateral ventricles.

## 4. Discussion

This work introduces a novel 3D quantitative aMRI (3D q-aMRI) post-processing algorithm that enables the visualization and quantification of the sub-voxel pulsatile brain motion in physical units. The algorithm is based on solving the optical flow equation over the phase coefficients of the 3D complex steerable pyramid decomposition and as such is a natural extension to the original 3D aMRI algorithm. By extending the original 3D aMRI to 3D q-aMRI and applying it to volumetric *in vivo* data, this method offers the capability to visualize and quantify the 3D piston-like motion of the brain and change in ventricular shape as the brain deforms through the cardiac cycle.

The phantom simulations suggest that when properly tuned, the 3D q-aMRI is capable of quantifying motion at a scale greater than 0.005 of a pixel size (the theoretical limit) for an SNR as low as 25. This is achieved while maintaining a high correlation (0.95), low error (13%), and a relatively small error distribution tail (99% of the error values fall below 30%). In general, phase-based methods, such as those used in 3D q-aMRI, are relatively robust to noise, as demonstrated by phantom simulations. However, it is important to note that when the data contain high levels of noise, the algorithm is likely to produce significant errors. In addition, in practice, there are likely confounding factors such as field inhomogeneity, partial volume effects, and various sources of ‘noise’, which were not comprehensively represented in the phantom simulation. These factors are likely to limit 3D q-aMRI’s ability to accurately estimate motions as small as 0.005 pixels in size. Yet, as demonstrated in Figure 8b, the motion profile observed in the cerebral aqueduct is continuous and smooth. This suggests that the algorithm has successfully detected coherent signal changes corresponding to voxel displacement at a scale of 0.01 pixels.

A noteworthy observation from the phantom simulation is that the algorithm performs very accurately, even for very low-contrast data, such as what is seen in the brain ventricles. As long as some level of texture exists in the data and the voxels exhibit subtle signal changes corresponding to motion, the algorithm appears capable of accurately estimating the voxel displacement field-even in the presence of considerable noise (low SNR). This claim comes with the condition that in a small neighborhood, the motion field remains relatively constant-as this factor influences the algorithm’s accuracy. Thankfully, this assumption seems valid for the human brain but will require further evaluation in cases that may present extreme brain motion patterns. It is worth mentioning that some texture almost always exists in the *in vivo* data. For example, the lateral ventricles, which are supposed to contain only CSF (same T1 and T2 property, and as such the same contract) contain some texture. Finally, the results suggest that 3D q-aMRI can adequately quantify motion across a range of temporal frequencies.

The study’s *in vivo* validation demonstrates that 3D q-aMRI successfully quantifies (in the form of a voxel displacement field) the observed signal in 3D aMRI. It is important to distinguish between two types of quantification: (1) quantification of the 3D aMRI unamplified signal and (2) comparison of 3D q-aMRI with true quantitative methods, such as PC-MRI. This validation procedure supports the first type of quantification. The algorithm hyperparameters were tuned empirically according to the phantom simulation observations. It is important to note that brain motion induced by other physiological signals, such as respiration and vasomotion, may require different hyperparameter tuning. However, for cardiac-induced motion, we observed that most of the motion occurs in the first two levels of the 3D steerable pyramid. This suggests that the pulsatile brain motion is composed of local deformations rather than global translation (rigid body motion). In addition, most of the motion is concentrated within the one to four heart rate harmonics band. This suggests that the cardiac input, which is periodic, contains higher harmonics in addition to the main heart rate (fundamental harmonic) and/or that the brain, as a nonlinear system, is excited by higher harmonic modes in response to the cardiac input. In addition, we noticed that for a Gaussian filter with σ>5, the estimated motion field profile is almost unchanged. This observation helps to determine a threshold for how much smoothing is needed to remove noise without oversmoothing the estimated field. This observation is similar to our previous work on 3D aMRI [36,37], where we visually observed that for a Gaussian filter with σ>5, the amplified data contain minimal noise, and the amplitude of the brain motion is smaller. Finally, we determined the range of image isotropic resolution (1.2–1.8 mm) for which the estimated motion field remains relatively consistent. Larger motion displacement was observed in low-resolution data. This is likely to be due predominantly to errors in low-resolution images, which are plagued by partial volume effects. Our analysis here emphasizes the importance of standardizing 3D q-aMRI for clinical applications, including various parameters, such as image resolution and dimension, Gaussian filter (σ value), and temporal filtering frequency band, which have an impact on the extracted brain motion field (mainly the amplitude). Notice that other true quantitative methods such as DENSE-MRI are also subject to variation in tissue displacement estimation as a function of image resolution [47].

It is worth mentioning that, like many other MRI techniques, 3D q-aMRI is prone to rigid body motions, which can be categorized into coherent and non-coherent motions. Noncoherent motion results in spatial blurring in the ‘cine’ MRI data, which in turn results in an error in the estimated voxel displacement field. Coherent motion-such as bulk head motion induced by blood pulsation-is detectable by 3D q-aMRI and can be corrected by subtracting the estimated voxel displacement field in the skull (which is assumed to be static) from the estimated displacement field.

There is an important limitation of 3D q-aMRI that must be highlighted. The algorithm is unable to estimate motion accurately beyond 1.5 pixels. This is primarily because small motions are assumed for the linearization of the phase constancy equation (Equation (8)). When dealing with heavily pulsating vessels like the basilar artery, which can move through several pixels in a cine dataset, these dynamic structures can introduce errors in the estimated motion field. This underscores the importance of thoroughly examining both the raw and amplified data in conjunction with the displacement field maps.

Previous research has illustrated the intricate nature of the brain’s biomechanics, showing the interplay between the CSF and the pulsatile brain motion. The general characteristics of pulsatile brain motion and the estimated voxel displacement field extracted by 3D q-aMRI resemble the displacement field extracted by PC-MRI and other studies [5,6]. The predominant displacement of tissue and fluid occurs in the cranial–caudal direction in the sagittal and coronal planes. Additionally, the axial plane shows expansion/contraction motion, and the most significant tissue displacement is concentrated around the midbrain, brainstem, cerebellar tonsil, and hypothalamus regions. Due to the reduced perfusion of brain tissue toward the cranium boundary, minimal displacement is observed in the frontal lobe, parietal lobe, occipital lobe, temporal lobe, and posterior cerebellum.

It is noteworthy that our choice of the 3D q-aMRI hyperparameters resulted in a motion/flow profile through the cerebral aqueduct, which matched the profile previously reported by [46], which used PC-MRI. While this is a promising finding that supports the current choice of the hyperparameters used here, the 3D q-aMRI flow profile was an order of magnitude (mm/s) smaller than that reported in the literature [46,48]. This is an interesting finding. Although 3D q-aMRI does not directly measure CSF flow like PC-MRI, it may provide complementary information about tissue compliance alongside flow measurements. It seems like the cerebral aqueduct pulsatile motion may result from the pressure of the CSF flowing through it- and that regional brain displacement and CSF flow may be tightly coupled. This observation suggests that 3D q-aMRI may be capable of detecting the CSF pulsatile flow profile in various brain compartments, such as the subarachnoid and perivascular space. Again, while the flow profile itself is not quantifiable, it could lead to an important method of indirectly assessing CSF homeostasis, and further testing should be performed to confirm this.

The *in vivo* validation of our study underscores the potential of 3D q-aMRI to serve as a reliable quantitative tool in clinical studies, as it can generate ’heat maps’ of pulsatile brain motion, which may assist in assessing disease-induced biomechanical differences. Our study observed that the most substantial brain motion occurs in the superior–inferior direction, which is consistent with previous studies. This motion is likely attributed to the pulsatile nature of the CSF flow, which is strongly influenced by the cardiac cycle.

Additionally, our results demonstrate that 3D q-aMRI is both repeatable and reproducible. The intra-correlation coefficient (ICC) analysis suggests significant differences between within-subject and across-subject measurements, indicating the potential to detect individual differences even within this healthy volunteer cohort. However, in some repeat scans, we did observe some variation in the amplitude of the voxel displacement profile (Supplemental Figure S6). Upon examining these ‘cine’ data, we identified global temporal flickering (Supplemental Video S9). The source of the flickering is still under investigation, but we suspect it may be related to the high acceleration factor (eight in our case) and the reconstruction scheme. So far, 3D q-aMRI has been applied to product sequences, such as the GE ’cine’ FIESTA. Our future work aims to jointly optimize the acquisition and reconstruction of 3D q-aMRI to further enhance its repeatability. It is also worth noting that heart rate exerts a significant influence on the estimated motion magnitude. We believe that this is a direct physiological consequence of cardiac pulsatility. Our data is cardiac-gated, and motion is assumed to result from blood flow and pulsations transferred through the larger arteries within a cardiac cycle. Hence, any alterations in the amplitude or frequency of these pulsations-such as natural heart rate variations-are likely to impact the timescale available for transferring these pulsations carried by the pulsatile blood flow. Monitoring the effects of heart rate variations during the scan and understanding their impact on data interpretation are topics that will be explored in future studies.

It is also important to recognize that while 3D q-aMRI demonstrated reliable detection of sub-voxel brain motion, other MRI techniques (CSPAMM, PC-MRI, and DENSE-MRI) are also capable of detecting this type of motion. With its high temporal resolution, high spatial resolution, good tissue contrast, and short scan time, 3D q-aMRI is a practical clinical alternative method. For example, the acquisition time for the PC-MRI data in this study was 4.5 min (for only three slices, with a single encoding direction, and with a much lower resolution of 1.9 × 1.9 × 4 mm), and currently, the total acquisition time for a DENSE-MRI scan in a 3T scanner with 2.2 mm isotropic resolution is 16.5 min [5]. In addition, with a longer scanning time (15 min), 3D q-aMRI can achieve 0.8 mm isotropic spatial resolution without compromising temporal resolution and SNR. For comparison, the acquisition time for DENSE to achieve this resolution will be more than twenty times longer. This advantage might enable the detection of pulsatile motion of small features such as the perivascular spaces.

However, it is important to highlight that 3D q-aMRI estimates the voxel displacement field based on the signal intensity temporal changes in bSSFP ‘cine’ MRI. While the measured signal in DENSE-MRI and PC-MRI corresponds to the genuine spin displacement, in bSSFP, there are other physiological sources that may impact the signal intensity and cause 3D q-aMRI to interpret it as motion (like any other optical flow method)-even if they do not necessarily correlate to true displacement. As such, the method should not be considered as a true displacement quantification method like DENSE-MRI. There is no doubt that the extracted voxel displacement field is highly correlated and resembles physical units with tissue displacement, as seen in our PC-MRI analysis and [46,48,49], but further work is also necessary to characterize the true physiology behind the 3D q-aMRI signal variation.

Preliminary data from our study indicate that 3D q-aMRI has the potential for identifying biomechanical differences induced by disease and could serve as a diagnostic tool for detecting early signs of neurodegenerative disorders. Our preliminary study suggests that abnormal pulsatile brain motion might correlate with changes associated with cognitive decline; however, it is important to note that these findings are based on a small number of healthy volunteers, and further studies with larger and more diverse populations are necessary to validate these results and establish the clinical utility of 3D q-aMRI.

## 5. Conclusions

This study introduces a novel 3D quantitative aMRI (3D q-aMRI) post-processing algorithm capable of visualizing and estimating voxel displacement field due to pulsatile brain motion in physical units. Applied to standard 3D cine data, the method benefits from a short scan time and fast processing time, making it practical for clinical use. 3D q-aMRI is both repeatable and reproducible for a given set of acquisition parameters. Although 3D q-aMRI does not directly measure the true physical displacement of spins, it provides an estimate of the field and offers valuable quantitative insights into the brain’s biomechanical response in both healthy and pathological conditions. This makes it a potentially useful tool for research and diagnosis.

## 6. Patents

A PCT patent application (PCT/US2024/027154) was applied based on this work.

## Figures and Tables

**Table 1:** Direction cosine for the axes of symmetry of 6 basis filters $B_{j}$, with geometry based on the vertices of the cuboctahedron.

*j*	$\alpha_{j}$	$\beta_{j}$	$\gamma_{j}$
1	$\frac{1}{\sqrt{2}}$	$\frac{1}{\sqrt{2}}$	0
2	$\frac{1}{\sqrt{2}}$	$-\frac{1}{\sqrt{2}}$	0
3	$\frac{1}{\sqrt{2}}$	0	$\frac{1}{\sqrt{2}}$
4	$-\frac{1}{\sqrt{2}}$	0	$\frac{1}{\sqrt{2}}$
5	0	$\frac{1}{\sqrt{2}}$	$\frac{1}{\sqrt{2}}$
6	0	$\frac{1}{\sqrt{2}}$	$-\frac{1}{\sqrt{2}}$

**Table 2:** Correlation, error estimation, and 99th percentiles of the error distribution as a function of SNR for motion magnitudes > 0.005 of a pixel size.

SNR	No Noise	200	100	50	25	12.5	6.25
Correlation	0.98	0.98	0.97	0.96	0.95	0.93	0.94
Error [%]	5.69	5.88	6.60	9.35	12.65	12.12	16.16
Percentile (99%)	18.13	19.18	19.09	27.31	30.26	38.81	48.16
Sigma	5	5	7.5	10	12.5	15	17.5

## Data Availability

No formal data sets were created as part of this manuscript.

## Supplementary Materials

The following supporting information can be downloaded at: https://www.mdpi.com/article/10.3390/bioengineering11080851/s1.
