# Supplementary material for: 3D Quantitative-Amplified Magnetic Resonance Imaging (3D q-aMRI)
Source: Bioengineering (Basel). 2024 Aug 20;11(8):851. doi: 10.3390/bioengineering11080851 (PMC11352018; doi:10.3390/bioengineering11080851)
Supplement: Supplementary file 1 [file bioengineering-11-00851-s001.zip › Supporting Information 3D q-aMRI/Captions - supplemental material.pdf]

Supplemental Figure S1 - The error of the estimated motion field as a function of the number of pyramid levels for two different motion types: global translation and local deformations.

Supplemental Figure S2 - The effect of varying standard deviations (σ ranging from) on the estimated motion field for SNR of 25.

Supplemental Figure S3 - The effect of temporal filtering on the estimated motion field over time for SNR of 25.

Supplemental Figure S4 - The extracted motion field over time for a wide range of temporal frequencies (1-4 Hz).

Supplemental Figure S5 - The extracted flow/motion profile through the cerebral aqueduct as extracted by 3D q-aMRI (left) for nonoptimal hyperparameters tuning (temporal frequency bands 0.75-1.25 Hz).

Supplemental Figure S6 - The average (over the different brain regions) pulsatile brain motion profile for all six subjects in the S/I direction for eight scans (t_0 to t_7). The black line is the average motion over all eight scans together with an error bar (95% confidence interval). Column 7 depicts the average motion (the black line) and error bar (95% confidence interval) for the six subjects. The last column is the correlation matrix of the average voxel displacement depicted in column 7.

Supplemental Video S1 – Phantom simulation results for SNR of 50. The 3D digital phantom was designed to mimic the subtle deformation, intensity, and contrast of the lateral ventricles observed in 3D aMRI. The original phantom (left), and the amplified extracted field (right). 3D q-aMRI successfully estimated the true motion field even in the presence of significant noise and very little contrast.

Supplemental Video S2 – Comparison between 3D aMRI and 3D q-aMRI. 3D q-aMRI successfully quantified the motion field observed in 3D aMRI.

Supplemental Video S3 – The amplified (3D aMRI) movies for different pyramid levels (levels 1-6), demonstrating that motion information mostly exists within the first two levels of the 3D steerable pyramid.

Supplemental Video S4 – The amplified (3D aMRI) movies for different temporal frequency bands (1-6Hz), demonstrating that motion information mostly exists within the 1-4Hz band.

Supplemental Video S5 – 3D q-aMRI results for varying Gaussian windows (σ ranging from 0 – 12.5). Increasing the σ value result in reduced noise in the estimated motion, but the field profile is almost unchanged for $\sigma > 5$.

Supplemental Video S6 – 3D q-aMRI results for different isotropic voxel sizes (1.2-3.0 mm). The extracted motion field remains consistent up to an isotropic voxel size of 1.8mm.

Supplemental Video S7 – The original 'cine' data with red arrow pointing to basal artery, which exhibits apparent motion (larger than 1.5 pixels).

Supplemental Video S8 – Comparison between PC-MRI (top) and 3D q-aMRI (bottom) for sagittal (S/I direction, white arrow), coronal (S/I direction, white arrow), and axial (L/R direction, white arrow) planes. The estimated field captures the relative brain tissue deformation over time and the physical change in shape of the ventricles by the relative movement of the surrounding tissues.

Supplemental Video S9 – The original 'cine' data which exhibit global flickering.

Supplemental Video S10 – The amplified videos (sagittal and axial planes) and voxel displacement maps (L/R direction, indicated by white arrow) for healthy control and mild cognitive impairment (MCI) due to Alzheimer's disease subjects. Red arrows indicate the regions where abnormal motion was detected.

Supplemental Video S11 – The amplified videos (sagittal and axial planes) and voxel displacement maps (L/R direction, indicated by white arrow) for healthy control and dementia due to Alzheimer's disease (AD) subjects. Red arrows indicate the regions where abnormal motion was detected.

Supplemental Video S12 – The amplified videos (sagittal and axial planes) and voxel displacement maps (L/R direction, indicated by white arrow) for healthy control and dementia due to Alzheimer's disease (AD) subjects. Red arrows indicate the regions where abnormal motion was detected.

Supplemental Video S13 –The amplified videos (sagittal and axial planes) and voxel displacement maps (L/R direction, indicated by white arrow) for healthy control and mild cognitive impairment (MCI) due to AD subjects. Red arrows indicate the regions where abnormal motion was detected.

Supplemental Video S14 – The amplified videos (sagittal and axial planes) and voxel displacement maps (L/R direction, indicated by white arrow) for healthy control and Lewy body dementia (LBD) subjects. Red arrows indicate the regions where abnormal motion was detected.
