# Supplementary figures and images for: 3D Quantitative-Amplified Magnetic Resonance Imaging (3D q-aMRI)
Source: Bioengineering (Basel). 2024 Aug 20;11(8):851. doi: 10.3390/bioengineering11080851 (PMC11352018; doi:10.3390/bioengineering11080851)

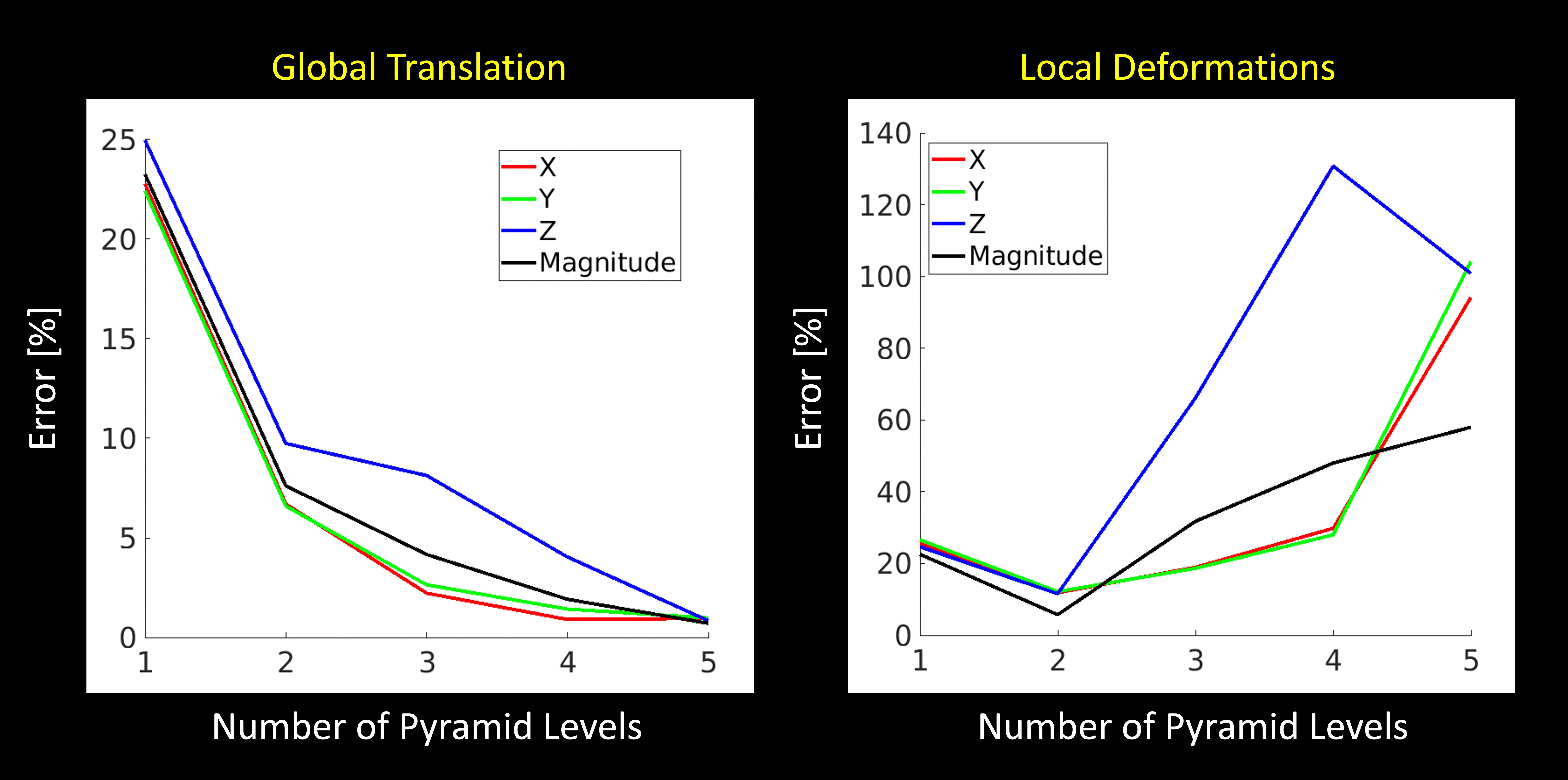

Supplement: Supplementary file 1 [file bioengineering-11-00851-s001.zip › Supporting Information 3D q-aMRI/figures/S1.jpg]

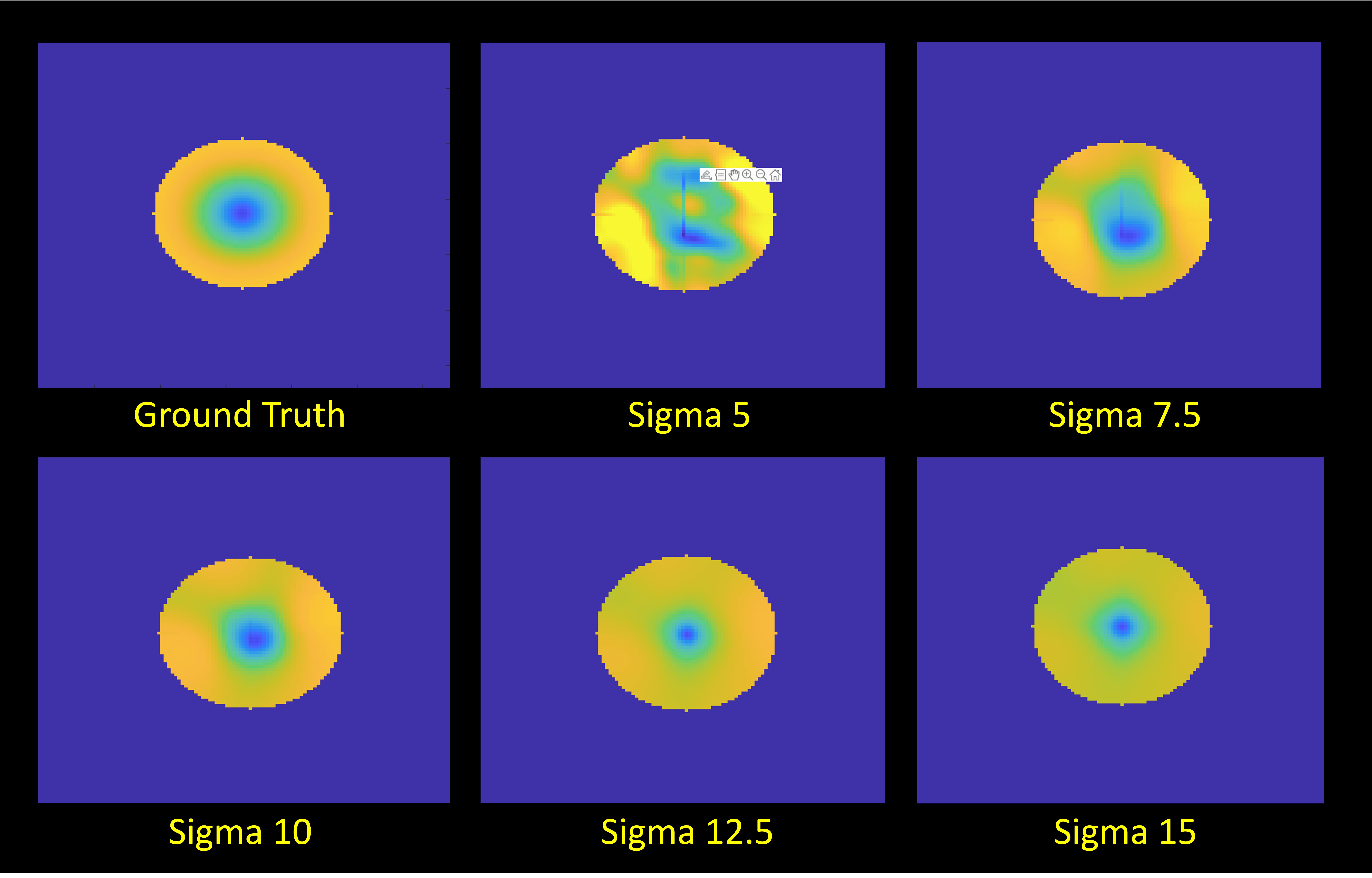

Supplement: Supplementary file 1 [file bioengineering-11-00851-s001.zip › Supporting Information 3D q-aMRI/figures/S2.jpg]

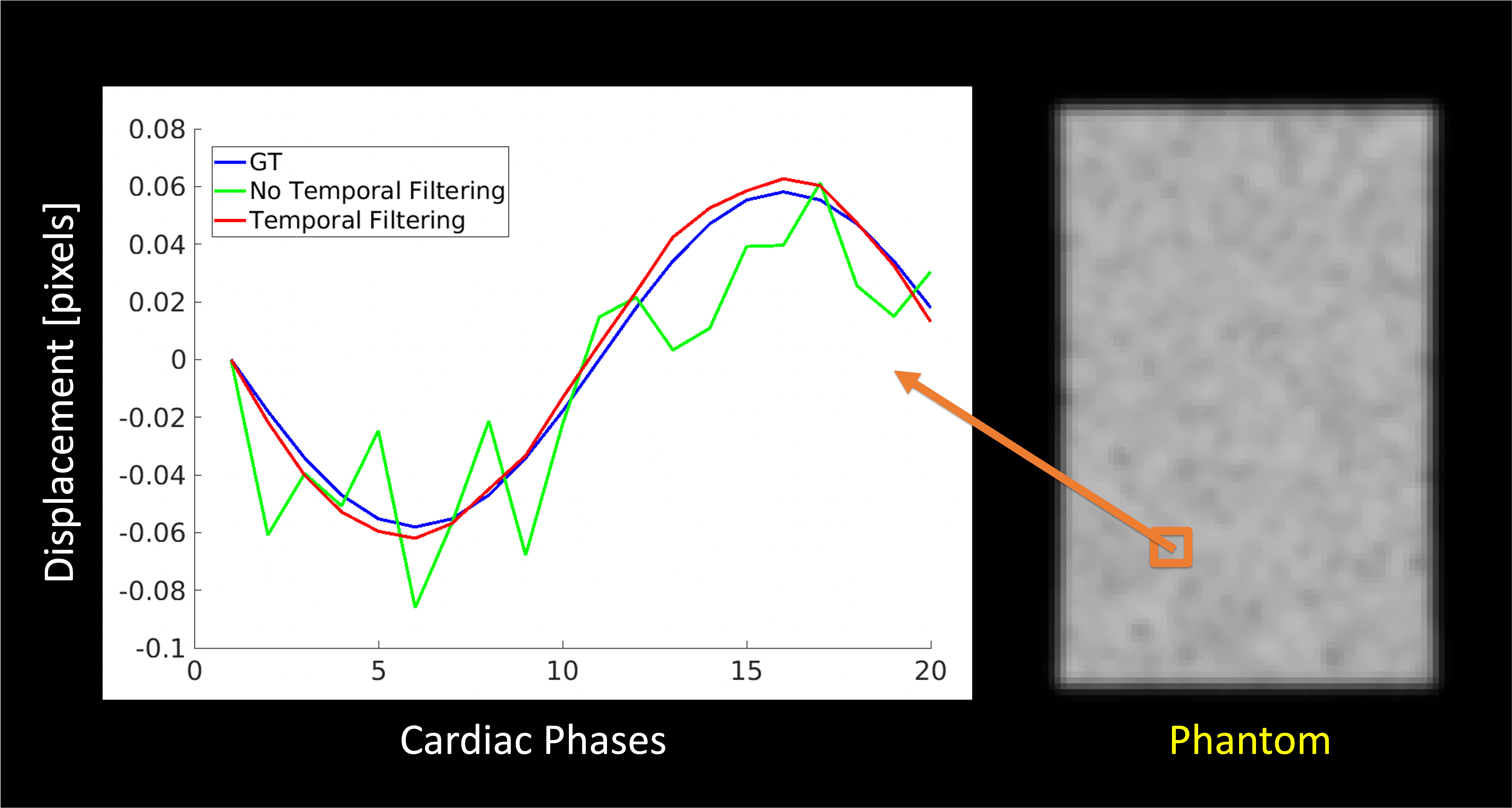

Supplement: Supplementary file 1 [file bioengineering-11-00851-s001.zip › Supporting Information 3D q-aMRI/figures/S3.jpg]

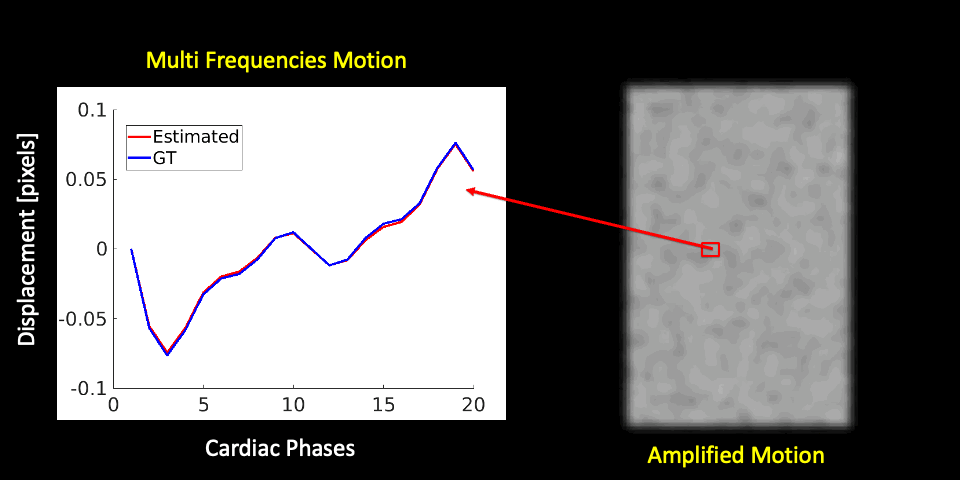

Supplement: Supplementary file 1 [file bioengineering-11-00851-s001.zip › Supporting Information 3D q-aMRI/figures/S4.gif]

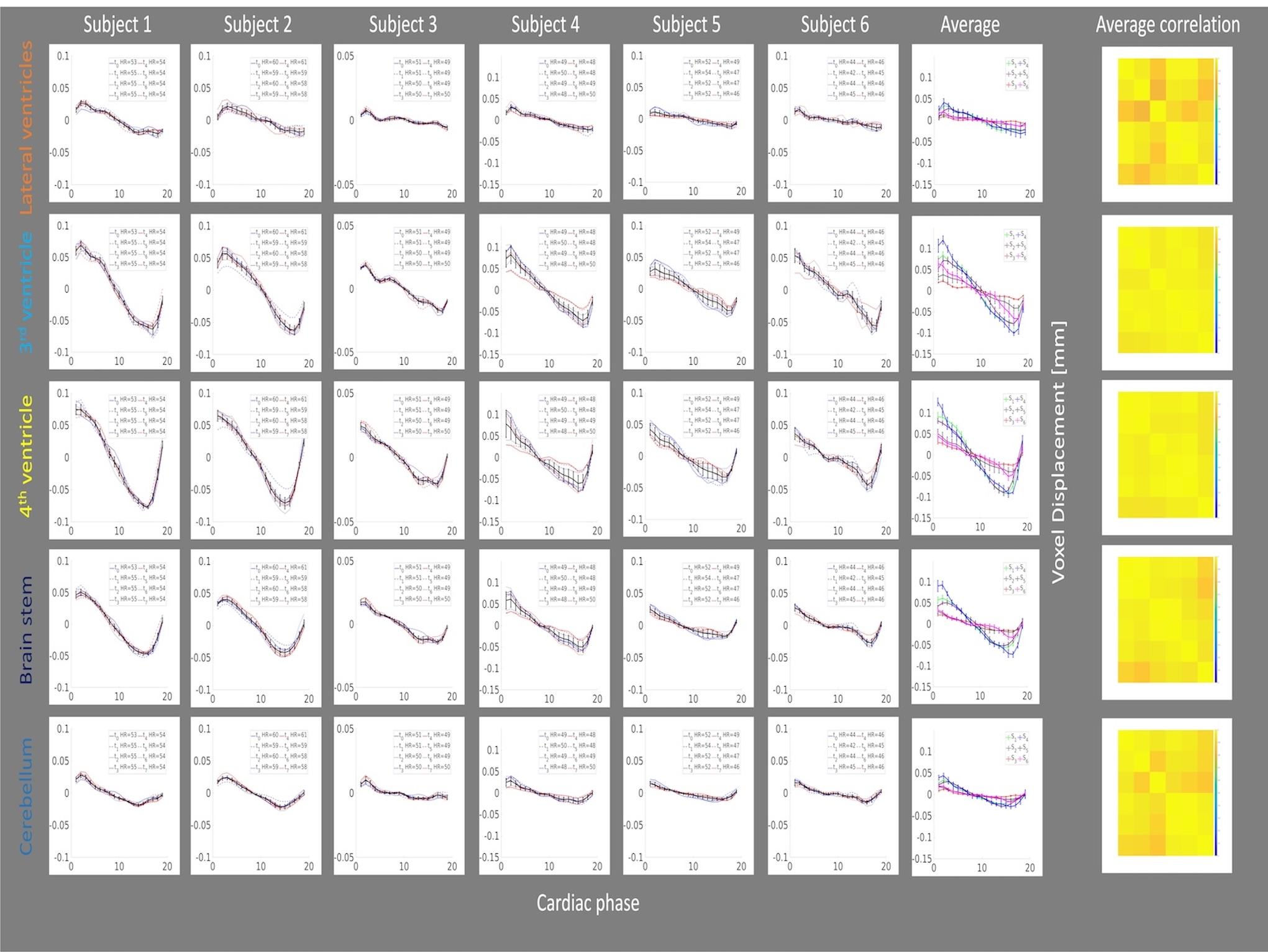

Supplement: Supplementary file 1 [file bioengineering-11-00851-s001.zip › Supporting Information 3D q-aMRI/figures/S6.jpg]
